# Systemic Inflammatory Response Index Is Associated With Insufficient Weight Loss After Bariatric Surgery

**DOI:** 10.1002/wjs.70151

**Published:** 2025-11-20

**Authors:** María Asunción Acosta‐Mérida, Raquel Bañolas‐Suárez, Marta Morera‐Sánchez, Joaquín Marchena‐Gómez

**Affiliations:** ^1^ Faculty of Health Sciences University of Las Palmas de Gran Canaria Las Palmas de Gran Canaria Canary Islands Spain; ^2^ Department of General and Digestive Surgery University Hospital of Gran Canaria Dr. Negrín Las Palmas de Gran Canaria Canary Islands Spain

**Keywords:** bariatric surgery, insufficient weight loss (IWL), obesity, systemic inflammation response index (SIRI)

## Abstract

**Background:**

Obesity is a chronic disease characterized by a persistent low‐grade inflammatory state. The systemic inflammatory response index (SIRI) is a novel biomarker of systemic inflammation. This study aimed to evaluate SIRI evolution before and 1 year after bariatric surgery and to assess its association with insufficient weight loss (IWL).

**Method:**

An observational study was conducted including all patients who underwent bariatric surgery at a university hospital between 2013 and 2023. Preoperative and 1‐year postoperative data were collected, including demographics, comorbidities, anthropometric parameters, surgical technique, complications, and SIRI values. Changes in these variables and their association with IWL were analyzed.

**Results:**

A total of 348 patients were included, 116 men (33.3%) and 232 women (66.7%), with a median age of 46 years (IQR 39–46). Sleeve gastrectomy was performed in 196 patients (56.3%) and Roux‐en‐Y gastric bypass in 152 (43.7%). Baseline weight (*p* < 0.001) and BMI (*p* = 0.005) were significantly associated with preoperative SIRI. Postoperative SIRI correlated with weight, BMI, and %EWL at 1 year (all *p* < 0.001). Higher postoperative SIRI values were significantly associated with IWL, both by %EWL < 50% (*p* = 0.008) and Reinhold's classification (*p* = 0.017). In multivariate analysis, higher SIRI at 1 year (*p* = 0.006; OR 0.27 and 95% CI 0.10–0.69), older age (*p* = 0.004; OR 0.96 and 95% CI 0.93–0.99), and female sex (*p* = 0.019; OR 0.52 and 95% CI 0.30–0.90) were independently associated with IWL.

**Conclusions:**

SIRI is closely associated with weight and BMI in patients with obesity and decreases after bariatric surgery, reflecting improved inflammatory status. Higher postoperative SIRI is independently associated with IWL, suggesting that persistent inflammation may contribute to poor surgical outcomes.

## Key Points

- SIRI is significantly associated with obesity severity both before and after bariatric surgery, highlighting its value as a marker of systemic inflammation in this population.
- SIRI decreases significantly 1 year after bariatric surgery, indicating a measurable improvement in inflammatory status following substantial weight loss.
- Postoperative SIRI levels are independently associated with insufficient weight loss (IWL), suggesting that persistent inflammation may play a role in suboptimal surgical outcomes.
- Age and SIRI were identified as independent predictors of IWL, supporting the potential role of SIRI in early risk stratification and postoperative monitoring.

## 1 Introduction

Patients with obesity exhibit a chronic low‐grade inflammatory state [1, 2, 3], which is closely associated with vascular and metabolic dysfunction. This persistent inflammation contributes to the development of cardiovascular comorbidities, type 2 diabetes mellitus, and even certain malignancies, that increase overall mortality risk in this population [4, 5].

The chronic inflammatory response in obesity is primarily mediated by dysregulated adipose tissue, which secretes proinflammatory cytokines and adipokines, such as tumor necrosis factor‐alpha (TNF‐α), interleukin‐6 (IL‐6), and leptin, while reducing anti‐inflammatory mediators such as adiponectin. These alterations generate a proinflammatory environment that exacerbates insulin resistance, endothelial dysfunction, and atherogenesis [6].

In recent years, attention has turned to the use of biomarkers that can indirectly assess this systemic inflammatory status. Simple hematological indices, such as the neutrophil‐to‐lymphocyte ratio (NLR), platelet‐to‐lymphocyte ratio (PLR), monocyte‐to‐lymphocyte ratio (MLR), the systemic immune‐inflammation index (SII), and the systemic inflammatory response index (SIRI), have emerged as reliable accessible indicators of systemic inflammation. These markers, easily derived from routine blood tests, have been associated with obesity‐related complications including metabolic syndrome, cardiovascular disease, and malignancy [7, 8, 9].

Bariatric surgery is one of the most effective interventions for severe obesity, producing substantial and sustained weight loss, improvement, or resolution of comorbidities, and enhanced quality of life. Although the metabolic benefits of bariatric surgery are well established, its long‐term effects on systemic inflammation and the behavior of inflammatory biomarkers remain less well understood. Furthermore, the relationship between postoperative inflammatory status and insufficient weight loss (IWL), observed in some patients after bariatric surgery, has yet to be clearly defined.

The aim of this study was to evaluate the evolution of the systemic inflammatory response index (SIRI) before and 1 year after bariatric surgery and to assess its association with insufficient weight loss.

## 2 Material and Methods

### 2.1 Study Design and Population

We conducted a retrospective cohort study analyzing data from consecutive patients who underwent primary bariatric surgery at a university hospital between January 2013 and March 2023.

### 2.2 Inclusion and Exclusion Criteria

Eligible patients were aged between 18 and 60 years and had a body mass index (BMI) ≥ 40 kg/m^2^ or a BMI ≥ 35 kg/m^2^ in the presence of obesity‐related comorbidities.

Exclusion criteria included the presence of psychiatric or eating disorders, substance abuse, incomplete medical records, prior bariatric procedures other than sleeve gastrectomy (SG) or Roux‐en‐Y gastric bypass (RYGB), and revisional bariatric surgery.

### 2.3 Ethics

The study was conducted in accordance with the principles of the Declaration of Helsinki and its subsequent updates. Approval was obtained from the Institutional Clinical Research and Ethics Committee (code 2023‐398‐1).

### 2.4 Management of the Patient

All patients underwent preoperative evaluation by a multidisciplinary team, including endocrinologists, nutritionists, psychologists, and psychiatrists, prior to surgical assessment.

Surgical intervention was indicated only after failure of lifestyle modification and conservative weight loss strategies. The preoperative work‐up included laboratory testing, upper gastrointestinal endoscopy, abdominal ultrasound, chest radiography, spirometry, and electrocardiogram. Written informed consent was obtained from all participants prior to surgery.

### 2.5 Surgical Procedure

All procedures were performed laparoscopically by experienced bariatric surgeons, following standardized institutional protocols.

For Roux‐en‐Y gastric bypass (RYGB), a 25–30 mL gastric pouch was created, followed by a linear side‐to‐side jejunojejunostomy and an antecolic side‐to‐side gastrojejunostomy, with a 200 cm alimentary limb and a 70 cm biliopancreatic limb.

Sleeve gastrectomy (SG) was performed using a 40‐Fr bougie for calibration, and the stomach was divided with a 60‐mm linear stapler, starting 4 cm from the pylorus up to the angle of his.

RYGB was preferred for patients with BMI > 40 kg/m^2^, metabolic syndrome, or gastroesophageal reflux disease. SG was favored in cases of malabsorption, iron deficiency anemia, inflammatory bowel disease, chronic diarrhea, advanced liver disease, immunosuppressive therapy, older age, or future pregnancy planning.

### 2.6 Follow‐Up

Postoperative clinical and laboratory follow‐up visits were scheduled at 1, 3, 6, 9, 12, 18, and 24 months postsurgery and annually thereafter. Patients were considered lost to follow‐up if no evaluation occurred within a 24‐month period.

### 2.7 Data Collection and Variables

Data were obtained from the institution's electronic medical records. The following variables were collected:

#### 2.7.1 Baseline Characteristics

Age, sex, comorbidity burden as measured by the Charlson Comorbidity Index [10], and preoperative risk classification according to the American Society of Anesthesiologists (ASA) [11].

#### 2.7.2 Anthropometric Data

Weight (kg) and body mass index (BMI) were recorded preoperatively and 1 year postoperatively. BMI was calculated as weight in kilograms divided by height in meters squared (kg/m^2^).

#### 2.7.3 Surgical Data

Variables included the type of bariatric procedure performed (Roux‐en‐Y gastric bypass [RYGB] vs. sleeve gastrectomy [SG]), length of hospital stay (in days), postoperative complications, reoperations, and postoperative mortality. Postoperative complications were classified using the Clavien–Dindo system: Grades I–II were considered minor complications, whereas grades III–IV were categorized as major complications [12, 13]. Postoperative mortality was defined as death occurring during hospitalization or within 30 days postsurgery.

#### 2.7.4 Inflammatory Status

The systemic inflammatory response index (SIRI) [14] was used to evaluate systemic inflammation. Neutrophil, lymphocyte, and monocyte counts were obtained from blood samples collected preoperatively and 1 year after surgery. SIRI was calculated using the following formula: (Neutrophil count × Monocyte count)/Lymphocyte count.

#### 2.7.5 Primary Outcome

The main outcome variable was insufficient weight loss (IWL) 1 year after surgery, defined using three complementary criteria:

1. *Percentage of Excess Weight Loss (%EWL)*: IWL was defined as %EWL < 50% [15]. To calculate the %EWL, the following formula was applied:
%EWL=[(Initialweight−Currentweight)/(Initialweight−Idealweight)]×100
  The *Devine formula* [16] was used to calculate the ideal weight for each patient:
$${\text{IBW}}_{\text{men}}=50+2.3\;\left(\text{height}\;\text{in}\;\text{cm}\;–\;152.4/2.54\right)$$
$${\text{IBW}}_{\text{women}}=45.5+2.3\;\left(\text{height}\;\text{in}\;\text{cm}\;–\;152.4/2.54\right)$$
2. *Reinhold classification* [17]: Patients with %EWL ≤ 50% and/or BMI ≥ 35 kg/m^2^ were classified as having IWL.
3. *Percentage of total weight loss (%TWL)*: The formula used for its calculation was as follows:
%TWL=[(Initialweight–currentweight)/Initialweight)]×100
  IWL was defined as %TWL < 20% [18, 19].

### 2.8 Statistical Analysis

Data were analyzed using SPSS software version 29.0 for Windows (IBM Corporation, Armonk, NY, USA) and lamovi software version 2.5.6 (The Jamovi Project, 2024).

#### 2.8.1 Descriptive Analysis

A descriptive analysis of the sample was first conducted. Categorical variables were presented as frequencies and percentages. Continuous variables were expressed as means and standard deviations (SD) if normally distributed or as medians and interquartile ranges (IQR) when non‐normally distributed. The Kolmogorov–Smirnov test was used to assess the normality of quantitative variables.

#### 2.8.2 Univariate Analysis

Univariate analyses were performed to evaluate: (i) differences in all variables according to the surgical technique employed (SG vs. RYGB); (ii) the association of baseline weight and BMI with the preoperative SIRI score, as a marker of preoperative inflammatory status; (iii) differences between preoperative and postoperative values for anthropometric and inflammatory parameters; and (iv) which predictor variables, including inflammatory markers, were associated with IWL. Proportions were compared using the chi‐squared test or Fisher's exact test as appropriate. Continuous variables were compared using the Mann–Whitney *U* test or Spearman's correlation test. The Friedman test was applied to paired data with non‐normal distributions.

#### 2.8.3 Multivariate Analysis

A multivariate analysis of IWL, defined as a dichotomous outcome (%EWL ≥ 50% vs. < 50%), was performed. Variables that were significant in the univariate analysis of IWL, as well as other clinically relevant variables (such as surgical procedure and sex), were included in the model even if they were not significant in the univariate analysis, in order to strengthen the robustness of the model. Due to skewed distributions, the SIRI score was log‐transformed (log_10_) before inclusion. A two‐sided *p*‐value < 0.05 was considered statistically significant.

## 3 Results

### 3.1 Baseline Characteristics

Of the initial 425 patients, 348 met all inclusion criteria and completed at least 1 year of postoperative follow‐up. The remaining patients were excluded due to one or more of the following reasons: lack of 1‐year follow‐up, absence of a blood sample at the 1‐year mark, or the presence of an acute inflammatory episode at the time of blood sampling. In some cases, data could not be retrieved because follow‐up was conducted in peripheral hospitals outside our institutional network.

The cohort consisted of 116 men (33.3%) and 232 women (66.7%) (*p* < 0.001), with a median age of 46 years (IQR: 39–53). The median age‐adjusted Charlson Comorbidity Index was 2.0 (IQR: 1.0–3.0). Regarding surgical risk, 2 patients (0.6%) were classified as ASA I, 99 (28.4%) as ASA II, 246 (70.7%) as ASA III, and 1 patient (0.3%) as ASA IV. Sleeve gastrectomy was performed in 196 patients (56.3%) and Roux‐en‐Y gastric bypass (RYGB) in 152 patients (43.7%). No postoperative complications were observed in 280 patients (80.5%). Mild complications (Clavien–Dindo grades I–II) occurred in 48 patients (13.8%), while 20 patients (5.7%) experienced moderate‐to‐severe complications (grades III–IV). The anastomotic leak (fistula) rate was 2.6% (nine patients). A total of 24 patients (6.9%) required reoperation. No operative mortality was reported. The median length of hospital stay was 3 days (IQR: 3.0–4.3).

Table 1 presents the comparative analysis between patients who underwent SG and those who underwent RYGB.

**TABLE 1:** Comparative analysis of patients undergoing SG versus RYGB.

	SG	Bypass	*p*
	*n* = 196 (56.3%)	*n* = 152 (43.7%)	
Age, median (IQR)	46.0 (39.0–52.0)	46.0 (39.0–52.0)	0.686
Sex			0.032*
Men	56 (28.6)	60 (39.5)	
Women	140 (71.4)	92 (60.5)	
ASA			0.004*
I–II	69 (35.2)	32 (21.1)	
III–IV	127 (64.8)	120 (78.9)	
Charlson score, median (IQR)	2.0 (1.0–3.0)	2.0 (1.0–3.0)	0.166
Diabetes mellitus			0.915
No	103 (52.6)	79 (52.0)	
Yes	93 (47.4)	73 (48.0)	
Clavien–Dindo score			0.009*
0	169 (86.2)	111 (73.0)	
I–II	19 (9.7)	29 (19.1)	
III–IV	8 (4.1)	12 (7.9)	
Dehiscence			0.481
No	192 (98.0)	147 (96.7)	
Yes	4 (2.0)	5 (3.3)	
Reoperation			0.054
No	187 (95.4)	137 (90.1)	
Yes	9 (4.6)	15 (9.9)	
Postoperative mortality	0 (0.0)	0 (0.0)	—
Preop weight, median (IQR)	127.5 (114.0–145.0)	140.0 (122.9–155.6)	< 0.001*
Postop weight (1 year), median (IQR)	87.0 (77.0–100.0)	93.8 (81.0–107.0)	0.014*
Preop BMI, median (IQR)	45.6 (41.6–52.8)	49.1 (45.0–53.9)	< 0.001*
Postop BMI (1 year), median (IQR)	31.2 (28.1–36.0)	33.1 (29.0–37.1)	0.096
Preop SIRI, median (IQR)	1.05 (0.78–1.62)	1.19 (0.88–1.70)	0.143
Postop SIRI (1 year), median (IQR)	0.70 (0.51–1.03)	0.82 (0.59–1.20)	0.016*
%EWL			0.827
No IWL (≥ 50%)	142 (72.4)	106 (69.7)	
Yes IWL (< 50%)	54 (27.6)	46 (30.3)	
Reinhold score			0.611
No IWL	134 (68.4)	100 (65.8)	
Yes IWL	62 (31.6)	52 (34.2)	
%TWL			0.984
No IWL (≥ 20%)	183 (93.4)	142 (93.4)	
Yes IWL (< 20%)	13 (6.6.0)	10 (6.6)	

Abbreviations: %EWL, percentage excess weight loss; IQR, interquartile range; IWL, insufficient weight loss; TWL, percentage total weight loss.

^*^ Statistically significant.

### 3.2 Anthropometric Trends

The median baseline weight was 133.5 kg (IQR: 117.6–151.8), and the median baseline BMI was 47.6 kg/m^2^ (IQR: 43.0–53.0). One year after surgery, the median weight decreased to 89.0 kg (IQR: 78.0–103.0) (*p* < 0.001) and the median BMI to 31.6 kg/m^2^ (IQR: 28.7–36.3) (*p* < 0.001). Table 1 shows the differences observed according to the surgical technique performed.

The median postoperative percentage of excess weight loss (%EWL) was 58.1% (IQR: 46.2–69.9). According to this criterion, 111 patients (31.9%) had a %EWL < 50% and were therefore classified as having insufficient weight loss (IWL), whereas 237 patients (68.1%) achieved a %EWL ≥ 50%.

Using the Reinhold classification, 114 patients (32.8%) met criteria for IWL and 234 patients (67.2%) did not.

The median percentage of total weight loss (%TWL) was 31.4% (IQR: 25.1–37.7). According to this parameter, 27 patients (7.8%) had a %TWL < 20% and were classified as IWL, whereas 321 patients (92.2%) had a %TWL ≥ 20%.

IWL, assessed by %EWL, Reinhold score, and %TWL, did not differ significantly between the two surgical procedures (Table 1).

Univariate analyses of IWL (Table 2) showed that the variables age (*p* < 0.001), postoperative weight (*p* < 0.001), preoperative BMI (*p* < 0.001), postoperative BMI (*p* < 0.001), and postoperative SIRI (*p* = 0.007) were significantly associated with IWL.

**TABLE 2:** Univariate analysis of IWL (%EWL ≥ 50 vs. < 50%).

	No IWL (%EWL ≥ 50)	IWL (%EWL < 50)	*p*
	*n* = 248 (71.3%)	*n* = 100 (28.7%)	
Age, median (IQR)	49.0 (40.5–56.0)	45.0 (39–51)	< 0.001*
Sex			0.065
Men	90 (36.3)	26 (26.0)	
Women	158 (63.7)	74 (74.0)	
ASA			
I–II	75 (30.2)	26 (26.0)	
III–IV	173 (69.8)	74 (74.0)	
Charlson score, median (IQR)	2.0 (1.0–2.0)	2.0 (1.0–3.0)	0.190
Diabetes mellitus			0.083
No	137 (55.2)	45 (45.0)	
Yes	111 (44.8)	55 (55.0)	
Surgical procedure			0.579
GV	142 (57.3)	54 (54.0)	
Gastric bypass	103 (42.7)	46 (46.0)	
Clavien–Dindo score			0.537
0	200 (80.6)	80 (80.0)	
I–II	32 (12.9)	16 (16.0)	
III–IV	16 (6.5)	4 (4.0)	
Dehiscence			0.456
No	240 (96.8)	99 (99.0)	
Yes	8 (3.2)	1 (1.0)	
Reoperation			0.375
No	229 (92.3)	95 (95.0)	
Yes	19 (7.7)	5 (5.0)	
Postoperative mortality	0 (0.0)	0 (0.0)	—
Preop weight, median (IQR)	132.0 (116.0–149.9)	137.0 (118.0–155.0)	0.127
Postop weight (1 year), median (IQR)	85.0 (73.8–95.0)	105.5 (91.5–119.0)	< 0.001*
Preop BMI, median (IQR)	46.5 (42.3–51.9)	49.9 (44.5–57.1)	< 0.001*
Postop BMI (1 year), median (IQR)	30.0 (27.5–33.5)	38.5 (34.8–42.3)	< 0.001*
Preop SIRI, median (IQR)	1.12 (0.80–1.62)	1.13 (0.80–1.78)	0.995
Postop SIRI (1 year), median (IQR)	0.71 (0.51–1.04)	0.85 (0.61–1.31)	0.007*

Abbreviations: %EWL, percentage excess weight loss; IQR, interquartile range; IWL, insufficient weight loss; TWL, percentage total weight loss.

^*^ Statistically significant.

### 3.3 Inflammatory Status Evolution

The median baseline SIRI was 1.1 (IQR: 0.8–1.7), which decreased to 0.8 (IQR: 0.5–1.1) 1 year after surgery. This reduction was statistically significant (*p* < 0.001).

### 3.4 Associations Anthropometric Parameters and Inflammatory Status

#### 3.4.1 Baseline Status

Univariate analysis revealed a statistically significant association between baseline weight and baseline SIRI (*p* < 0.001). Similarly, baseline BMI was significantly correlated with baseline SIRI (*p* = 0.005).

#### 3.4.2 Postoperative Status

**FIGURE 1:**
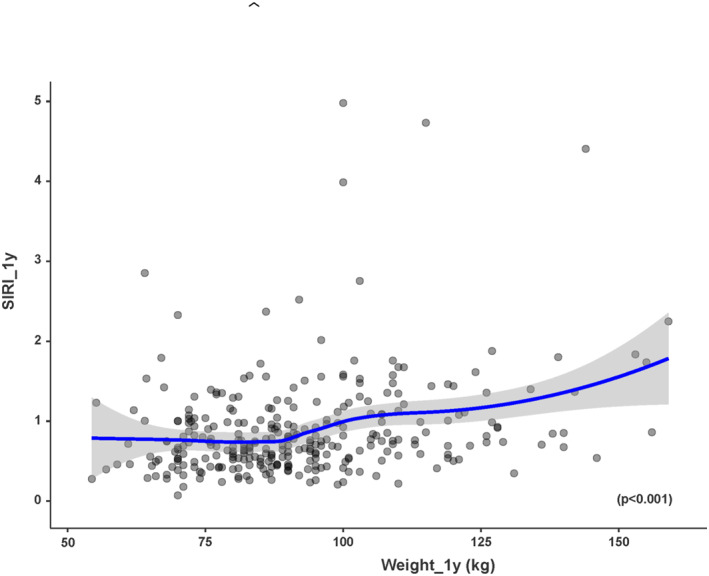
Correlation (Rho Spearman) between weight and inflammatory status (SIRI score) 1 year after surgery. The results indicate a positive correlation between weight and SIRI score, with higher weight values corresponding to higher SIRI‐scores (*p* < 0.001).

**FIGURE 2:**
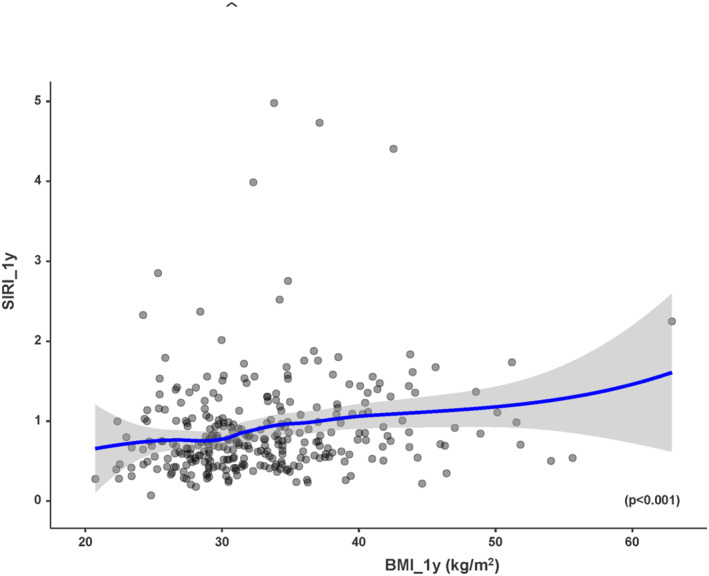
Correlation (Rho Spearman) between BMI and inflammatory status (SIRI score) 1 year after surgery. The results indicate a positive correlation between BMI and SIRI‐score, with higher BMI values corresponding to higher SIRI‐scores (*p* < 0.001).

Postoperative SIRI was significantly associated with postoperative weight (*p* < 0.001), postoperative BMI (*p* < 0.001), and percentage of excess weight loss (%EWL) (*p* < 0.001) (Figures 1 and 2). Higher postoperative SIRI values were correlated with greater weight and BMI and with lower %EWL.

Regarding the variable IWL, the SIRI score was significantly associated with the presence of IWL when assessed using %EWL (< 50% vs. ≥ 50%) (*p* = 0.007) and according to Reinhold's classification (*p* = 0.017).

Although a significant correlation was observed between SIRI and %TWL as a continuous variable (*p* = 0.016), this association was not significant when %TWL was categorized using the < 20% versus ≥ 20% cutoff (*p* = 0.606).

#### 3.4.3 Multivariate Analysis

**FIGURE 3:**
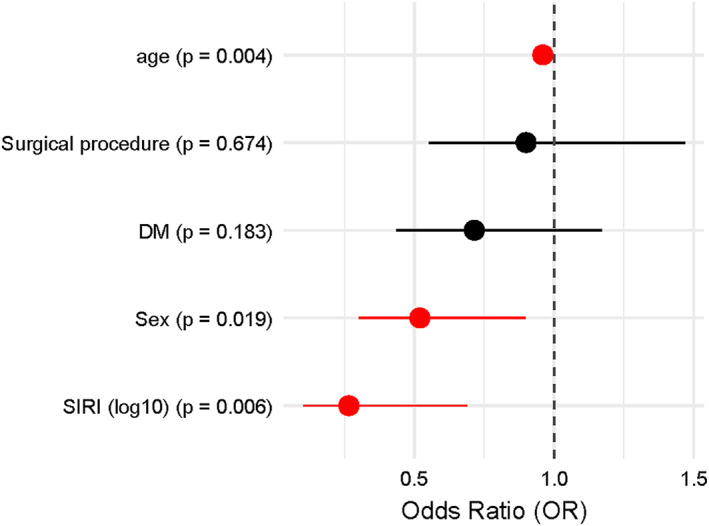
Forest plot of multivariate analysis (logistic regression) of IWL (%EWL ≥ 50 vs. %EWL < 50).

In the multivariate model, we included clinically relevant variables (such as surgical technique and sex), even if not significant in the univariate analysis, to strengthen the robustness of the model. The result is shown in Figure 3. On the other hand, we excluded baseline BMI from the primary model because %EWL already incorporates baseline weight/BMI in its calculation. Adjusting for initial BMI would introduce mathematical coupling and double adjustment for the same construct, potentially biasing estimates. Higher postoperative SIRI at 1 year (*p* = 0.006; OR: 0.27 and 95% CI: 0.10–0.69) and age (*p* = 0.004; OR: 0.96 and 95% CI: 0.93–0.99) were independently associated with IWL. Sex also showed a modest association (*p* = 0.19; OR: 0.52 and 95% CI: 0.30–0.90). Surgical procedure (RYGB vs. SG) and diabetes mellitus were not independently associated with the outcome (Figure 3).

## 4 Discussion

This study examined the potential relationship between the systemic inflammatory response index (SIRI) and obesity as well as postoperative weight outcomes following bariatric surgery. The key findings can be summarized as follows:

First, a significant association was observed between SIRI levels and the degree of obesity, both preoperatively and postoperatively.

Second, higher SIRI values were consistently associated with greater obesity severity.

Third, patients with insufficient weight loss (IWL) after bariatric surgery exhibited higher postoperative SIRI levels as demonstrated by both univariate and multivariate analyses.

These results suggest that SIRI may serve as a valuable indicator of systemic inflammation associated with obesity and could be related to suboptimal weight loss after bariatric surgery.

Previous studies have demonstrated that weight reduction in patients with obesity is usually accompanied by a decrease in inflammatory biomarkers [20, 21, 22, 23]. This improvement in inflammatory status appears to be more consistent when weight loss exceeds 10% [24], a threshold commonly used in bariatric surgery follow‐up [25].

C‐reactive protein (CRP), a well‐established biomarker of meta‐inflammation, has been shown to correlate strongly with weight, BMI, waist and hip circumferences, and waist‐to‐hip ratio [6]. Postbariatric surgery, CRP levels decrease significantly, both in the short and long term, across procedures such as sleeve gastrectomy [26, 27, 28], gastric bypass [29, 30], and adjustable gastric banding [30].

Similarly, obesity is associated with elevated white blood cell and neutrophil counts, which correlate directly with BMI [31, 32, 33]. Studies have reported that, in patients undergoing bariatric surgery, circulating lymphocyte and neutrophil levels decrease in proportion to the reduction in BMI over time [31]. Consistent with these findings, our results support the hypothesis that bariatric surgery has a beneficial effect on systemic inflammation [34]. Moreover, our study advances the field and points toward the possibility of using SIRI—a composite inflammatory index as a biomarker of inflammatory burden and weight loss in bariatric patients.

SIRI integrates neutrophils (markers of acute inflammation), monocytes (precursors of macrophages involved in immune defense and tissue remodeling), and lymphocytes (key regulators of immune response). As with the systemic immune‐inflammation index (SII) [35], SIRI offers a more holistic view of the inflammatory response than isolated ratios such as NLR, MLR, or PLR. Unlike individual cell counts, composite indices are less susceptible to fluctuations due to fluid status or transient physiological changes.

Several studies have demonstrated the prognostic utility of SIRI. For example, Xia et al. [8] found that individuals with SIRI > 1.43 had significantly higher risks of all‐cause (HR 1.39 and 95% CI: 1.26–1.52) and cardiovascular mortality (HR 1.39 and 95% CI: 1.14–1.68) compared to those with SIRI < 0.68. Both SIRI and SII have been associated with metabolic and inflammatory diseases such as hepatic steatosis, diabetes, dyslipidemia, and metabolic syndrome [36, 37, 38, 39, 40].

In a large cohort study involving over 20,000 individuals [41], including 7890 with obesity, high SIRI and SII levels were significantly associated with obesity. Interestingly, the relationship followed a nonlinear dose‐dependent pattern, indicating a complex interaction between inflammation and adiposity. These findings suggest that different BMI and inflammatory thresholds may be required when evaluating biomarker performance in this context [41].

Furthermore, Liu et al. [42] reported that SIRI—but not SII—was an independent predictor of cardiovascular disease prevalence in obese individuals. SIRI and SII have also been shown to independently predict all‐cause and cardiovascular mortality, with SIRI demonstrating superior predictive power [9]. These data support the use of SIRI as a clinically meaningful biomarker for evaluating inflammatory status and therapeutic response in obesity [9].

To our knowledge, this is the first study to demonstrate an association between SIRI and weight loss outcomes following bariatric surgery. These findings support the potential of SIRI as a marker for identifying patients at risk of suboptimal surgical response (“non‐responders”) and tailoring postoperative management strategies accordingly.

However, one of the main drawbacks of these studies is the current lack of consensus on the definition of insufficient weight loss after bariatric surgery, with similar variability observed in the criteria used to define weight regain [19]. This lack of standardization introduces methodological heterogeneity and limits comparability across studies. In our study, we did not include weight nadir as a criterion. Instead, we used %EWL < 50% [15] and the Reinhold classification [17], which combines %EWL and BMI as more appropriate metrics. Moreover, we observed that the commonly used TWL < 20% cutoff may be inadequate as SIRI correlated with TWL as a continuous variable but not with the dichotomized threshold. We believe that this cutoff point should be revised in larger patient cohorts. This reinforces the need to revisit current definitions and establish standardized criteria.

Our study also found that older age was independently associated with IWL, consistent with previous literature indicating that older patients tend to have poorer weight loss outcomes following bariatric surgery compared to younger individuals [43, 44].

### 4.1 Limitations

The main limitations of this study include its retrospective design, the long follow‐up period, and the potential for unmeasured confounding variables. Nevertheless, the large sample size, uniform surgical protocols, and single‐center design enhance the internal validity and generalizability of the findings.

## 5 Conclusions

The main findings of this study showed that:

1. SIRI is a reliable biomarker of systemic inflammation, strongly associated with weight and BMI in patients with obesity.
2. Bariatric surgery leads to a significant reduction in SIRI, reflecting an overall improvement in inflammatory status.
3. Higher postoperative SIRI levels are significantly associated with insufficient weight loss, suggesting that persistent inflammation is related to poor surgical response.

Further prospective studies are needed to clarify the causal relationship between systemic inflammation and weight loss outcomes and to validate the use of SIRI as a prognostic and therapeutic tool in bariatric populations.

## Author Contributions

**María Asunción Acosta‐Mérida:** conceptualization, methodology, project administration, supervision, validation, visualization, data curation, writing – original draft, writing – review and editing. **Raquel Bañolas‐Suárez:** data curation, writing – original draft, writing – review and editing. **Marta Morera‐Sánchez:** data curation, investigation, writing – review and editing. **Joaquín Marchena‐Gómez:** formal analysis, methodology, validation, writing – original draft, writing – review and editing, supervision.

## Funding

The authors have nothing to report.

## Ethics Statement

The study was conducted in accordance with the principles of the Declaration of Helsinki and its subsequent updates. Approval was obtained from the Institutional Clinical Research and Ethics Committee (code 2023‐398‐1).

## Consent

The requirement for written informed consent was waived because of the retrospective design of this study.

## Conflicts of Interest

The authors declare no conflicts of interest.

## Data Availability

Data will be made available upon reasonable request from the corresponding author.
